# Transvaginal fast-scanning optical-resolution photoacoustic endoscopy

**DOI:** 10.1117/1.JBO.23.12.121617

**Published:** 2018-12-05

**Authors:** Yuan Qu, Chiye Li, Junhui Shi, Ruimin Chen, Song Xu, Hasan Rafsanjani, Konstantin Maslov, Hannah Krigman, Laura Garvey, Peng Hu, Peinan Zhao, Karen Meyers, Emily Diveley, Stephanie Pizzella, Lisa Muench, Nina Punyamurthy, Naomi Goldstein, Oji Onwumere, Mariana Alisio, Kaytelyn Meyenburg, Jennifer Maynard, Kristi Helm, Janessia Slaughter, Sabrina Barber, Tracy Burger, Christine Kramer, Jessica Chubiz, Monica Anderson, Ronald McCarthy, Sarah K. England, George A. Macones, Qifa Zhou, K. Kirk Shung, Jun Zou, Molly J. Stout, Methodius Tuuli, Lihong V. Wang

**Affiliations:** a Washington University in St. Louis, March of Dimes Prematurity Research Center, Department of Obstetrics and Gynecology, St. Louis, Missouri, United States; b Washington University in St. Louis, Department of Biomedical Engineering, St. Louis, Missouri, United States; c California Institute of Technology, Caltech Optical Imaging Laboratory, Andrew and Peggy Cherng Department of Medical Engineering and Department of Electrical Engineering, Pasadena, California, United States; d University of Southern California, NIH Ultrasonic Transducer Resource Center, Department of Biomedical Engineering, Los Angeles, California, United States; e Texas A&M University, Department of Electrical and Computer Engineering, College Station, Texas, United States; f Washington University in St. Louis, Department of Pathology and Immunology, St. Louis, Missouri, United States

**Keywords:** optical-resolution photoacoustic endoscopy, fast scanning, microelectromechanical system scanning mirror, cervical imaging

## Abstract

Photoacoustic endoscopy offers *in vivo* examination of the visceral tissue using endogenous contrast, but its typical B-scan rate is ∼10  Hz, restricted by the speed of the scanning unit and the laser pulse repetition rate. Here, we present a transvaginal fast-scanning optical-resolution photoacoustic endoscope with a 250-Hz B-scan rate over a 3-mm scanning range. Using this modality, we not only illustrated the morphological differences of vasculatures among the human ectocervix, uterine body, and sublingual mucosa but also showed the longitudinal and cross-sectional differences of cervical vasculatures in pregnant women. This technology is promising for screening the visceral pathological changes associated with angiogenesis.

Vasculatures enable nutrient transportation, waste disposal, and immune surveillance. Due to the diverse functions of blood vessels, abnormally morphological vascular changes are often associated with the development of various diseases, including tumor growth and metastasis,^1^^–^^3^ inflammatory disorders, and pulmonary hypertension,^4^ to name just a few. Many models linking vascular morphogenesis to the development of a particular disease have been developed for prognosis, diagnosis, or disease management.^5^ To apply these models in clinical assessment, however, a tissue biopsy from the lesion is needed. Because tissue biopsy is invasive and often not clinically feasible,^6^ technologies that provide *in vivo* noninvasive examination of vascular networks are clinically useful,^7^ but generally do not have enough resolution and specificity to resolve microcirculation vessels.

Optical-resolution photoacoustic microscopy using endogenous optical absorption contrast enables *in vivo* vascular imaging with a capillary-level spatial resolution, and it has emerged as a major tool for inspecting morphological changes in the vascular network.^8^^–^^11^ Photoacoustic endoscopy (PAE), by miniaturizing the tabletop setup of photoacoustic microscopy, can reach organs in body cavities and noninvasively acquire visceral vascular images.^12^^–^^14^ These endoscopic devices use rotary scanners, which enable a large angular field of view, up to ∼310  deg, but also constrain the B-scan rate to ∼10  Hz. As a result, motion artifacts due to the natural *in vivo* movement of tissue (breathing movement, peristalsis, etc.) often appear in the endoscopic images. Thus, the development of new PAE systems with higher imaging speeds is a top priority to broaden its clinical application. Microelectromechanical system (MEMS) scanning mirrors have demonstrated their superior scanning speed, high accuracy, and simple system design in various biomedical imaging modalities.^15^^,^^16^ A water-immersible version with a small footprint has the potential to increase the imaging speed of PAE by an order of magnitude.

Here, we present a fast-scanning optical-resolution PAE (fsOR-PAE) that uses a custom-designed MEMS scanning mirror. The B-scan rate can reach 250 Hz over a 3-mm range, faster than the previous device by a factor of 10.^13^ This imaging speed allowed us to acquire visceral vascular images in humans *in vivo*, with a volumetric imaging speed of 0.75 Hz.

Figure 1 is a schematic of our fsOR-PAE system. The system [Fig. 1(a)] is controlled by a custom-designed program written in LabVIEW (National Instruments). A fiber laser (V-Gen, VPFL-G-20) operates at 532-nm wavelength with a 500-kHz pulse repetition rate. We control the laser pulse energy by tuning a variable neutral density filter so that the optical fluence on the tissue surface is $∼17\;\mathrm{mJ}/{\mathrm{cm}}^{2}$, below the American National Standards Institute safe exposure limit.^17^ A photodetector (Thorlabs, PDA36A) detects the light reflected from the neutral density filter, and a voltage comparator connected to the photodetector generates a trigger for each laser pulse to synchronize the whole system. The laser beam is spatially filtered by a 50-μm diameter pinhole (Thorlabs, P50CH) before being coupled into a single-mode optical fiber (Thorlabs, S405-XP). The fiber guides the light to the fsOR-PAE probe with an insertion tube 20 cm in length and 20 mm in diameter [Fig. 1(b)].

**Fig. 1:**
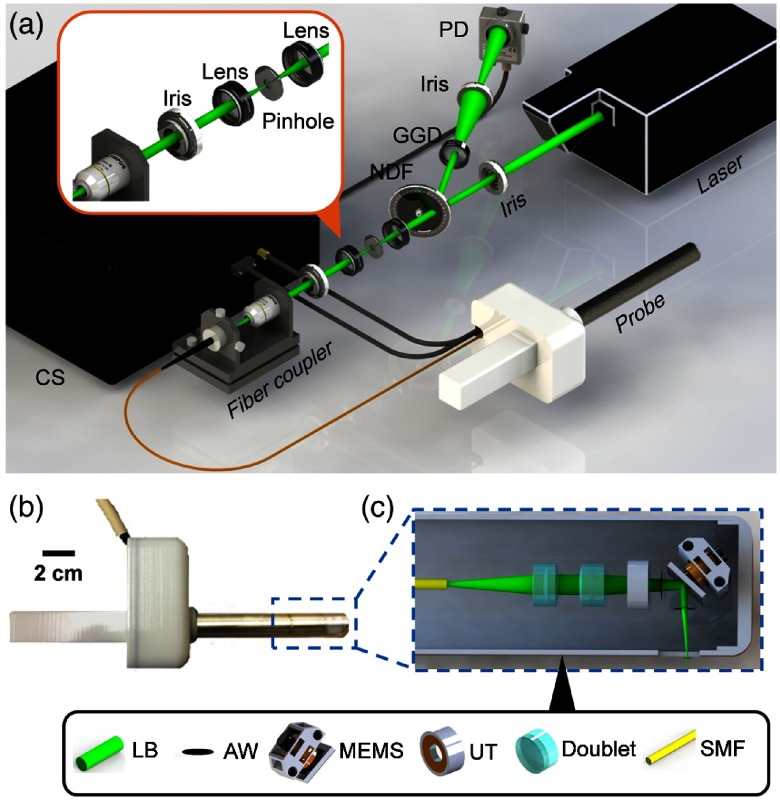
Schematic of the fsOR-PAE probe and its peripheral systems. (a) Setup of fsOR-PAE. CS, control system; GGD, ground glass diffuser; NDF, variable neutral density filter; PD, photodetector. (b) Photograph of the fsOR-PAE probe. A linear actuator in the white housing drives the azimuth scanning. (c) Schematic of the acoustic-optical coaxial confocal alignment in the probe. MEMS drives the scanning parallel to the cylindrical axis. AW, acoustic wave; LB, laser beam; MEMS, microelectromechanical system scanning mirror; SMF, single-mode fiber; UT, ultrasonic transducer.

In the probe, the laser beam from the single-mode fiber is focused by a set of doublets (Thorlabs, AC064-015-A) and then transmitted through the center of a custom-designed focused ultrasonic ring transducer (40-MHz central frequency), achieving an acoustic-optical coaxial confocal alignment [Fig. 1(c)]. To optimize the optical and acoustic transmittances through the imaging window, a polymethylpentene membrane (CS Hyde, 33-3F-24) seals the imaging window, preventing leakage when the chamber of the probe is filled with distilled water for ultrasound coupling. A MEMS scanning mirror drives the focal spot scanning parallel to the cylindrical axis of the probe [Fig. 2 (Video 1)]. It has two hinges (0.75 mm in length, 0.5 mm in width, and 0.2 mm in thickness) supporting a mirror plate (7 mm in length, 5 mm in width, and 1 mm in thickness), which consists of a polished silicon substrate, an aluminum reflective layer (200 nm), and a ${\mathrm{SiO}}_{2}$ protective overcoat (20 nm). The fast scanning is attributed to the oscillation of the mirror plate: a pair of permanent magnets fixed on the back of the mirror plate oscillate around the hinges in response to a sinusoidal current applied to the static inductor coil (inductance: 1  μH) in the MEMS device. The azimuthal scanning, with a step size of 3  μm, is driven by a linear actuator (Haydon Kerk, 21F4AC-2.5) in the white housing of the probe [Fig. 2 (Video 1)].

**Fig. 2:**
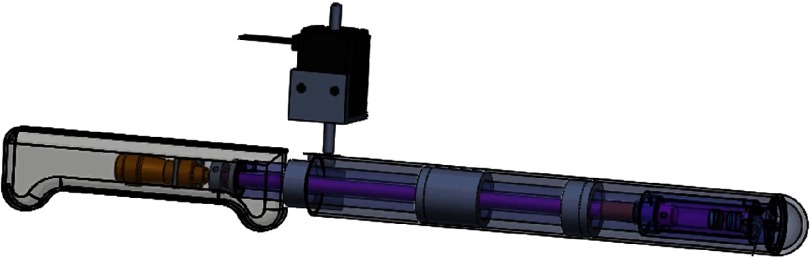
Scanning mechanism of the fsOR-PAE probe (Video 1, MP4, 3.78 MB [URL: https://doi.org/10.1117/1.JBO.23.12.121617.1]).

To test the performance of the fsOR-PAE system, we imaged tissue-mimicking phantoms. The lateral resolution of fsOR-PAE was quantified by imaging the edge of a sharp blade. From the edge spread function, the line spread function was computed and found to have a full width at half maximum of 3.1  μm, which represents the lateral resolution [Fig. 3(a)]. The axial resolution was estimated to be 46.5  μm, based on the photoacoustic signal detected from a tungsten wire (diameter: 14  μm) [Fig. 3(b)]. A metal grid (127-μm pitch and 37-μm bar width) [Fig. 3(c)] was imaged [Fig. 3(d)], and the average signal-to-noise ratio (SNR) was 33.2 dB. Figure 3(e) is a B-scan image in the plane highlighted by the dashed line in Fig. 3(d). Because the angular scanning of fsOR-PAE maps the detected photoacoustic signal in polar coordinates, we transform the data to Cartesian coordinates in image reconstruction.^16^ These results suggest that the fsOR-PAE system is capable of imaging structures on the micrometer scale.

**Fig. 3:**
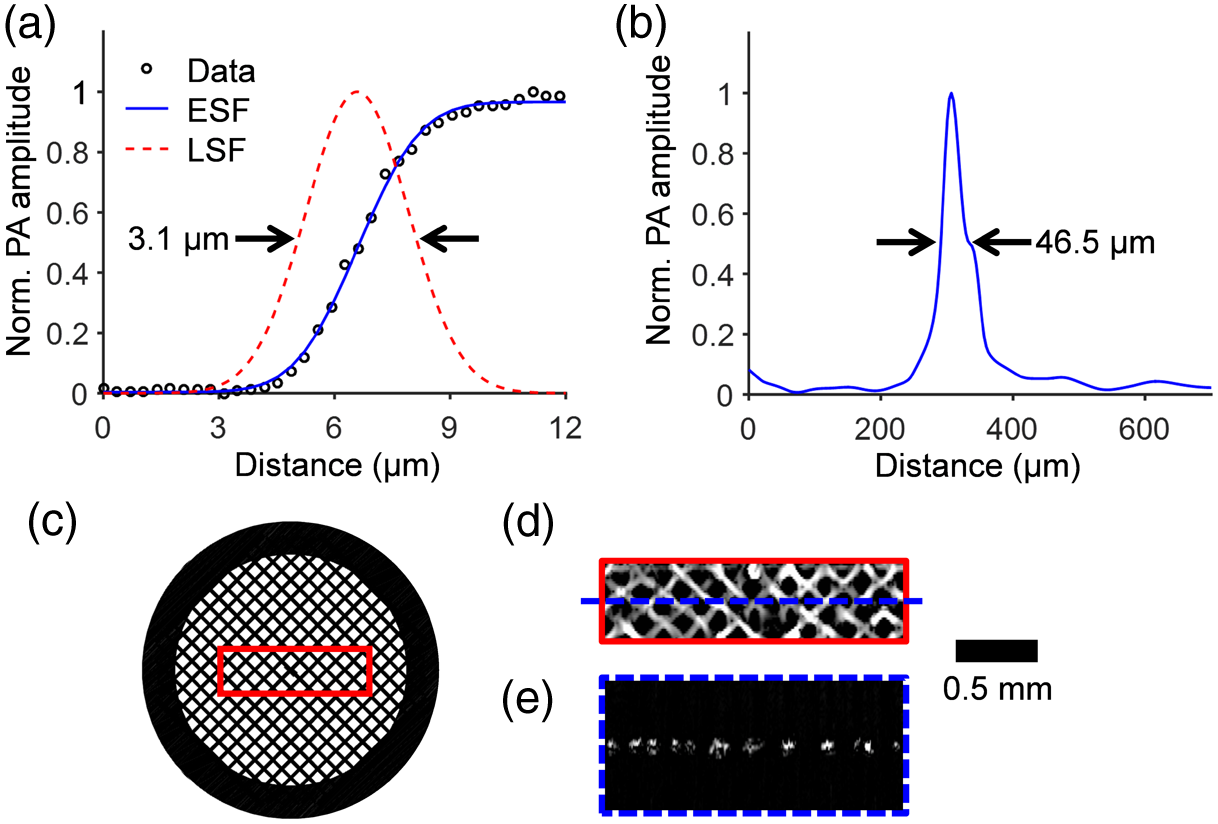
Characterization of the fsOR-PAE probe. (a) Lateral resolution test on a sharp edge. ESF, edge spread function; LSF, line spread function derived from ESF. (b) Axial resolution test on a tungsten wire. (c) Photograph of a metal grid. (d) Maximum amplitude projection image computed from the region enclosed by the red rectangle in (c). (e) B-scan image in the plane highlighted by the blue dashed line in (d).

We then imaged various human tissues to demonstrate the imaging capability of fsOR-PAE. All the human experiments followed protocols approved by the Institutional Review Board administered by the Human Research Protection Office at Washington University in St. Louis.

In an *ex vivo* demonstration, we imaged a uterus obtained from hysterectomy. Figures 4(a) and (b) show the vascular networks in the ectocervix and the serosal layers of the uterine body, respectively. A volume-rendered image is shown in Fig. 5 (Video 2). Viewed as a projection on the coronal plane, the blood vessels in the ectocervix are more likely to have a small aspect ratio and to be oriented toward the sagittal plane. In addition, the morphology of the vascular network clearly varies from one tissue to another. For example, blood vessels longer than 2 mm are absent in the ectocervix [Fig. 4(a)], but these long blood vessels can be easily found in the human sublingual mucosa [Fig. 4(c)]. Additionally, we carefully investigated the imaged tissue to demonstrate the safety of fsOR-PAE. Standard hematoxylin and eosin stain on the imaged area after fsOR-PAE imaging showed no evidence of tissue damage, necrosis, or heat injury [Fig. 4(d)].

**Fig. 4:**
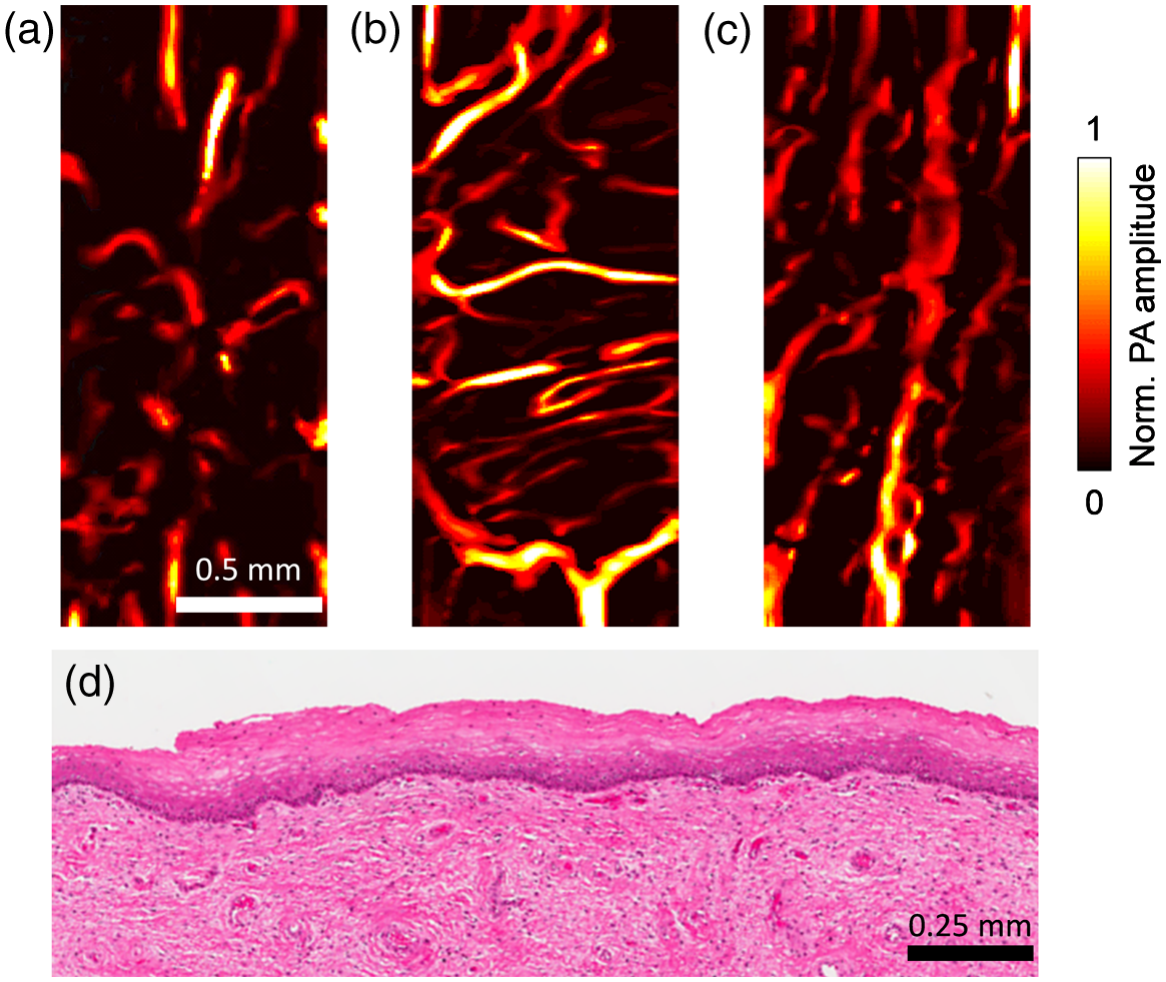
*Ex vivo* fsOR-PAE images of (a) the human ectocervix, (b) the serosal layer of the uterine body, and (c) the sublingual mucosa. (d) Standard hematoxylin and eosin histology of the ectocervix conducted after fsOR-PAE imaging, showing no tissue damage.

**Fig. 5:**
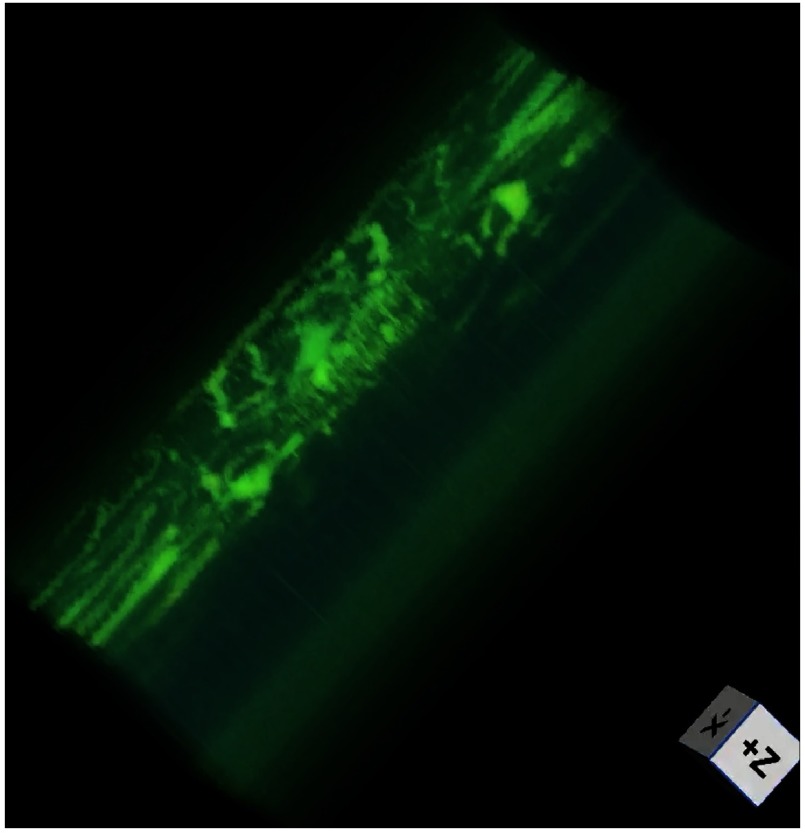
Volume-rendered image (Video 2, MP4, 890 KB [URL: https://doi.org/10.1117/1.JBO.23.12.121617.2]).

After we validated the imaging capability and safety of fsOR-PAE, we tested this imaging modality *in vivo* on human subjects. Previous studies found that cervical remodeling during pregnancy was associated with increased vascularity.^18^^,^^19^ We enrolled (n=2) pregnant women and imaged the anterior surface of the ectocervix for our study. The first pregnant woman was imaged at 32 weeks of gestation [Fig. 6(a)] and again at 36 weeks of gestation [Fig. 6(b)]. In this subject, we did not observe a perceptible change of vascular aspect ratio or blood vessel orientation over this time frame. This patient is 30 years old, had two prior deliveries, and had an operative vaginal delivery for nonreassuring fetal status at 39 weeks of gestation in our study. We imaged a second subject to perform a between-subject comparison and noted that blood vessels in the ectocervix of subject 2 had a smaller aspect ratio than in subject 1 at the same gestational age (36 weeks) [Fig. 6(c)]. This patient is 24 years old, had one prior delivery, and labored at 38 weeks of gestation in our study.

**Fig. 6:**
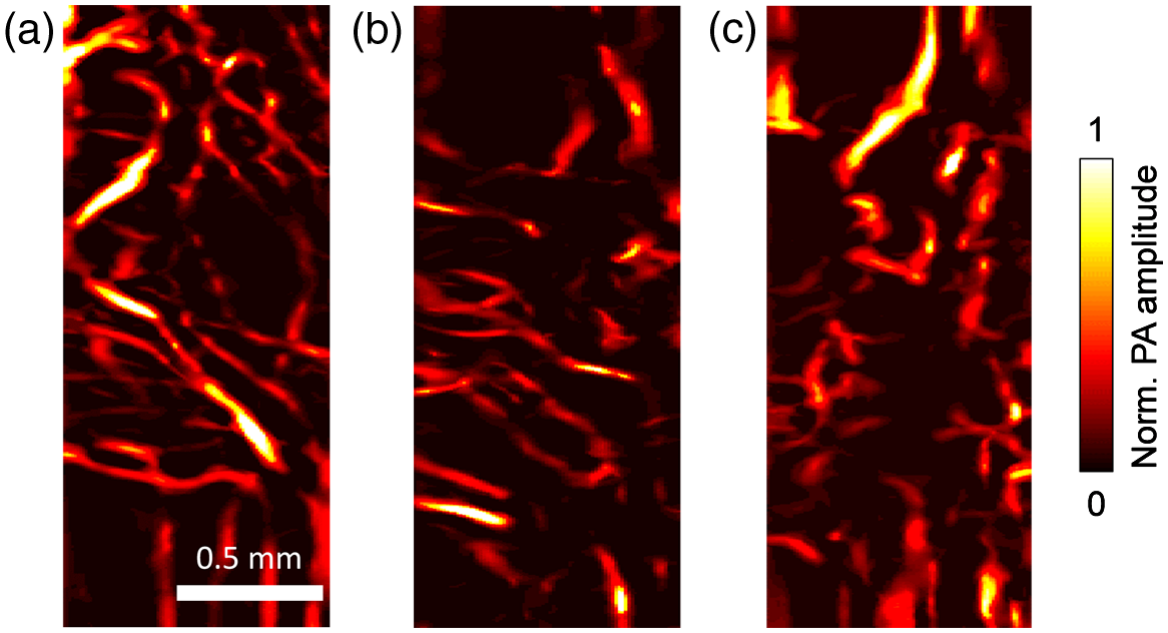
*In vivo* fsOR-PAE images acquired from the first pregnant woman at (a) 32 and (b) 36 weeks of gestation. (c) *In vivo* fsOR-PAE image acquired from the second pregnant woman at 36 weeks of gestation.

To explore what physiological features can be quantified from the fsOR-PAE images, we extrapolate from the two vascular parameters^5^ that could have close relationships with cervical remodeling: (1) the microvessel density (the number of vessels per unit area) and (2) total microvascular area (the percentage of area occupied by blood vessels) as shown in Fig. 7. Each parameter was calculated from five images measured from different areas. In the analysis, the blood vessels were segmented in three-dimensional (3-D) space, using a threshold set at three times the noise level, estimated as the standard deviation of the background signal outside the imaged region. The segmented outcomes were visually inspected and corrected if necessary. Our results show that the microvessel density is the more promising parameter for identifying the progress of cervical remodeling [Fig. 7(a)]. The total microvascular area, however, is a more discriminatory parameter for classifying the type of tissue [Fig. 7(b)]. Of course, these conclusions require validation in larger, blinded preclinical studies.

**Fig. 7:**
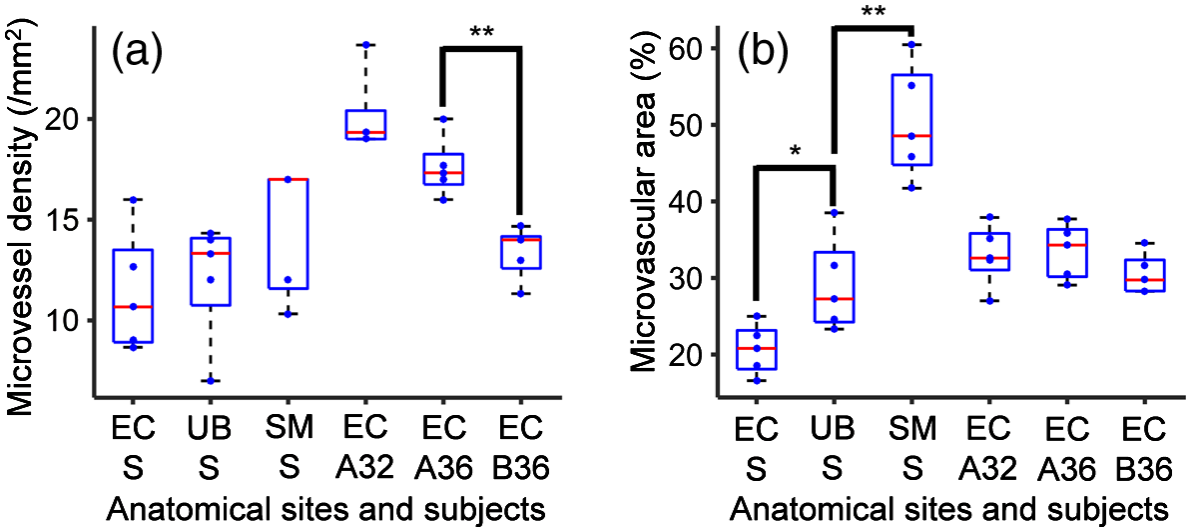
Box plots for the histomorphological quantities calculated from the fsOR-PAE images. Five images were analyzed for each subject. (a) Microvessel density and (b) total microvascular area. EC A32, the ectocervix of the first patient at 32 weeks of gestation; EC A36, the ectocervix of the first patient at 36 weeks of gestation; EC B36, the ectocervix of the second patient at 36 weeks of gestation; EC S, the ectocervix specimen; SM S, the sublingual mucosa specimen; UB S, the uterine body specimen. ^*^P<0.05, ^**^P<0.01.

In summary, we have developed an fsOR-PAE system that can achieve a 250-Hz B-scan rate over a 3-mm scanning range. This research presents the first high-resolution *in vivo* imaging of the vascular network in the human cervix, and its capillary-level spatial resolution is beyond the scope of current clinical methods.^7^ Further improvements could include minimizing the size of the probe to reach smaller cavities in the human body and exploiting a dual-wavelength light source to quantify oxygen metabolism.^20^ Furthermore, with the development of artificial intelligence, emerging classification models may divulge latent information which is beyond human recognition, but more valuable for diagnosis than the conventional histomorphological quantities in the fsOR-PAE images.^21^^,^^22^
